# Determinants of prehospital lactate in trauma patients: a retrospective cohort study

**DOI:** 10.1186/s12873-020-00314-1

**Published:** 2020-03-11

**Authors:** E. ter Avest, J. Griggs, J. Wijesuriya, M. Q. Russell, R. M. Lyon

**Affiliations:** 1 Air Ambulance Kent, Surrey and Sussex, Redhill Aerodrome, Redhill Airfield, Redhill, Surrey, RH1 5YP UK; 2 Department of Emergency Medicine, University Hospital Groningen, Groningen, the Netherlands; 3 School of Health Sciences, University of Surrey, Guildford, UK

**Keywords:** Lactate, Prehospital, Helicopter emergency medical service (HEMS)

## Abstract

**Background:**

Point of care serum lactate measurement is emerging as an adjunct to prehospital clinical assessment and has the potential to guide triage and advanced treatment decision-making. In this study we aimed to assess which factors potentially affect prehospital lactate levels.

**Methods:**

We performed a retrospective cohort study of all trauma patients attended by the Air Ambulance, Kent, Surrey & Sussex (AAKSS) between July 2017 and April 2018 in whom a pre-hospital lactate was measured. Lactate was measured before AAKSS treatments were commenced, but generally after prehospital treatment by ground ambulance crews was initiated. Primary endpoint of interest was the association of various patient- and treatment characteristics with prehospital lactate levels.

**Results:**

During the study period, lactate was measured in 156 trauma patients. Median lactate was 3.0 [2.0–4.1] mmol/l. Patients with an elevated lactate more often had deranged indices of end organ perfusion- and oxygenation (shock index 0.80 [0.58–1.03] vs 0.61 [0.40–0.82], *p* < 0.001, SpO_2_ 96 [89–100%] vs 98 [96–100%], *p* = 0.025). They more often suffered from head injuries (62% vs 41%, *p* = 0.008), and received less analgesia prior to arrival of the AAKSS team (51.6% vs 67.2%, *p* = 0.03). In multivariate analysis, indices of end organ perfusion- and oxygenation only explained 15% of the variation in lactate levels.

**Conclusions:**

Prehospital lactate levels are not solely associated with indices of end organ perfusion- and oxygenation. Injury type, treatments given on scene and many other (unmeasured) factors likely play an important role as well. This should be taken into account when lactate is used in clinical algorithms to guide prehospital triage or treatment.

## Background

Trauma triage guidelines are typically based on injury mechanism, injuries identified and vital signs reported. Reliance on vital signs and physical exam however, has been reported to miss patients with serious injury [1]. Point of care (POCT) serum lactate measurement is emerging as an adjunct to pre-hospital clinical assessment and has the potential to guide triage and advanced treatment decision-making: pre-hospital lactate levels have been shown to predict the need for resuscitative in-hospital care in trauma patients [2–5], and to predict outcome of trauma patients [6].

Historically, lactate formation in trauma patients was thought to originate from anaerobic glycolysis: hemorrhage and inadequate ventilation following a traumatic injury can lead to hypovalemia, hypoxaemia and end-organ hypoperfusion, resulting in anaerobic glycolysis and lactate formation. Over the past decade however, it has become clear that (beta adrenergic mediated) accelerated aerobic glycolysis is a major contributor to lactate formation under various conditions as well [7]. This is important to realize, as beta adrenergic stimulation in pre-hospital trauma patients is almost universally present as a result from pain and/or stress. Furthermore, many pharmacological and non-pharmacological) treatments on scene have the potential to modulate beta adrenergic stimulation, and thereby influence lactate levels. Although this will not affect the prognostic ability of elevated prehospital lactate levels as such (several studies have shown that the prognostic ability of lactate exceeds the prognostic ability of markers of end organ hypoperfusion) [2–5], it might influence the use of lactate as a marker to guide advanced prehospital treatments such as blood product transfusion [8].

Therefore, this study aims to examine which patient- and treatment factors are related to POCT lactate levels in prehospital trauma patients.

## Methods

### Study design and subjects

We performed a retrospective study of all trauma patients attended by Air Ambulance Kent, Surrey Sussex (AAKSS) between July 2017 (when lactate measurements became available to the service) and April 2018 (when the study protocol was completed). All patients in whom a pre-hospital lactate levels was measured, irrespective of their age, injury type, injury severity or outcome were included. Consecutive patients with non-traumatic pathology in whom a lactate was measured were excluded.

### Setting

AAKSS is a helicopter emergency medical service (HEMS) covering three counties in the southeast of England with a resident population of 4.5 million and transient population of up to 8 million. Two doctor/paramedic teams respond in helicopters or rapid response cars from one base and the service attends approximately 2000 patients per year. Most patients attended by the HEMS service are first seen by a ground ambulance crew and/or a critical care paramedic.

### Lactate measurement

Prehospital lactate measurements became available to AAKSS HEMS teams as an adjunct to clinical assessment in July 2017 using the NOVA StatStrip® Biomedical Xpress™ Point of care (POCT) Lactate Meter system [9, 10]. Current AAKSS Standard Operating Procedures (SOP), recommend lactate measurement in all patients with suspected major hemorrhage. Lactate was measured from venous blood, drawn in a 2 ml syringe during venapuncture or after insertion of an intravenous canula. Lactate was drawn and analyzed in the prehospital setting before EMS treatments (including transfusion of any blood products) was commenced, but generally after prehospital treatment by ground ambulance crews (such as circulatory support, analgesia, and haemostatic interventions) was initiated.

### Outcome measures

The primary outcome measure in this study was defined as the association of various patient- and treatment characteristics with (elevated) prehospital lactate levels. In accordance with previous studies [11, 12], elevated lactate was defined as a blood concentration > 2.5 mmol/l.

### Data acquisition

Patient demographics, mechanism- and nature of injuries, vital signs, POCT test results (including lactate), and treatments provided by the HEMS team and other EMS services are recorded by clinical crews in a dedicated electronic patient record (HEMSbase 2.0, Medic One Systems Ltd., UK). Data were retrieved from this electronic recorded for the purpose of this study, using a standardized proforma (See Supplementary file 1).

### Ethics

This project met National Institute for Healthcare Research (NIHR, UK) criteria for service evaluation and ethical review was therefore not conducted by an external body (such as an NHS research Ethics Committee [13]. All the data used for this study were routinely collected as part of standard prehospital and hospital patient data collection. The project was approved by the AAKSS Research & Development Committee. The study has been performed in accordance with the ethical standards laid down in the Declaration of Helsinki.

### Patient and public involvement

It was not appropriate or possible to involve patients or the public in the design, or conduct, or reporting, or dissemination of our research.

### Statistical analysis

Shapiro Wilk tests were performed to assess normal distribution, and residual plots were drawn to assess linearity of data. Descriptive statistics are given as numbers [%] or median [interquartile range, IQR]. Comparisons across groups were made using Fisher’s exact test and Mann-Whitney U test where appropriate. Univariate correlation analysis with calculation of Spearman correlation coefficients was performed to evaluate the correlation of clinical- and treatment factors with the primary outcome (lactate). Stepwise multivariable regression analyses including factors with a significant correlation was performed to determine which factors were independently related to the primary outcome measure. Missing values are reported in the results section of the manuscript according to the STROBE guideline [14]. A *p*-value < 0.05 was regarded as statistically significant. All statistical analyses were conducted using IBM SPSS 23.0 for Apple statistical package (SPSS Inc., Chicago, Illinois, USA) and VassarStats online statistical software (Vassarstats.net).

## Results

### Study population

During the study period, 1188 patients were seen by AAKSS. A prehospital lactate was obtained in 174 patients (14.6%). Eighteen patients were excluded as they were attended by HEMS for a medical (non-trauma) reason (supplementary file 1). Subsequent results refer to the remaining 156 trauma patients (13.1%). The majority of the patients were male (79.5%), and involved in road traffic collisions (blunt trauma mechanism, 89.7%). The average time from 999-call to drawing blood for a lactate sample was 66 [46–87] minutes. Injuries, vital signs upon arrival of HEMS, and treatments provided by the HEMS team or other emergency medical services before blood was drawn to determine lactate, are described in Table 1.

**Table 1:** Study population characteristics

	All (*n* = 156)	Lactate < 2.5 mmol/L (*n* = 61)	Lactate > 2.5 mmol/L (*n* = 95)	*p*	missing
**Biometric data**					
Age (y)	44 [24–64]	44 [23–66]	45 [27–63]	.642	
Male Gender (n,%)	124 [79.5]	48 [78.7]	76 [80]	.842	
**Injury characteristics**					
Mechanism					
Blunt trauma (n,%)	140 [89.7]	56 [91.8]	84 [88.4]	.595	
Sharp trauma (n,%)	16 [10.3]	5 [8.2]	11 [11.6]	.595	
Body regions affected					
Head (n,%)	84 [53.8]	25 [41.0]	59 [62.1]	.008	2
Chest (n,%)	73 [46.8]	31 [50.8]	42 [45.2]	.513	2
Abdomen (n,%)	72 [46.2]	29 [47.5]	43 [45.3]	.870	
Limb (n,%)	75 [48.1]	29 [47.5]	46 [48.4]	.990	
Nr of regions affected (n)	2 [0–4]	2 [0–4]	2 [0–4]	.373	
**Lactate**					
999-lactate sample (min)	66 [46–87]	62 [45–80]	67 [46–88]	.420	33
Lactate (mmol/l)	3.0 [2.0–4.1]	1.8 [0.5–2.2]	3.8 [2.7–4.9]	<.001	
**Indices of end organ perfusion and oxygenation**					
Palpable radial pulse [y]	138 [88.5]	58 [95.1]	80 [84.2]	.042	
SBP (mmHg)	129 [109–149]	134 [117–152]	123 [102–144]	.001	10
HR (bpm)	88 [66–110]	85 [64–106]	96 [75–117]	.023	14
Shock index	.72 [.51–.93]	.61 [.40–.82]	.80 [.58–1.03]	<.001	14
EtCO_2_ (kPa)	4.1 [3.5–5.4]	4.1 [2.0–6.0]	4.0 [3.2–4.8]	.390	44
SpO_2_ (%)	97 [93–100]	98 [96–100]	96 [89–100]	.025	16
**Treatments before HEMS**					
Circ and resp. support					
Adrenaline	6 [3.8]	1 [1.6]	5 [5.3]	.405	
IV fluids	14 [9.0]	2 [3.3]	12 [12.6]	.050	
Pain relieving interventions					
IVP and/or morphine	87 [55.8]	41 [67.2]	49 [51.6]	.031	
IVP	61 [39.1]	30 [49.2]	31 [32.6]	.045	
morphine	51 [32.7]	23 [37.7]	28 [29.5]	.299	
Reduction or splinting^a^	29 [18.6]	8 [13.1]	21 [22.1]	.207	
Haemostatic interventions					
Pelvic splint	76 [48.7]	28 [45.9]	48 [50.5]	.624	
Compression Bandage or tourniquet	14 [9.0]	7 [11.5]	7 [7.4]	.402	
TXA	75 [48.1]	26 [42.6]	49 [51.6]	.325	

*Footnote***:** Continuous data represented as n [%], nominal data as median [IQR]. ^a^manual reduction of fracture or dislocation and/or Kendrick and/or vacuum splint application. *SBP* Systolic blood pressure, *HR* Heart rate, *EtCO*_*2*_ End tidal CO_2_, *SpO*_*2*_ Percentage oxygen saturation, *IV* Intravenous, *IVP* Intravenous paracetamol, *TXA* Tranexamic acid.

### Correlation of patient- and treatment characteristics with lactate levels

Injury type was related to (elevated) lactate levels: Patients with head injury more often had elevated lactate levels (41% vs. 62%, *p* < 0.008, Table 1), and head injury was correlated to lactate levels (r = 0.22, *p* = 0.006, Table 2). As expected from previous studies, lactate levels were related to markers of end-organ perfusion- and oxygenation (radial pulse, heart rate [HR], systolic blood pressure [SBP], Shock index [SI], Oxygen saturation (SpO_2_) and end-tidal CO_2_ [EtCO_2]_) (Tables 1 and 2). SI demonstrated the highest correlation with lactate levels (r = 0.35, *p* < 0.001). Several treatment factors were related to lactate levels: iv fluid administration showed a (weak) positive correlation with lactate levels, whereas administration of IV analgesia, showed an inverse relation with both absolute- and elevated lactate levels (Table 2).

**Table 2:** Univariate correlation coefficients of patient characteristics with pre-hospital lactate levels in trauma patients attended by HEMS (*n* = 156)

	Lactate (mmol/l)		Lactate > 2.5 mmol/l	
	r [95%CI]	*p*	r [95% CI]	*p*
**Biometric data**				
Age (y)	.01 [−.15–.16]	.974	−.04 [−.24–.17]	.643
Gender (n,%male)	−.03 [−.18–13]	.758	−.02 [−.17–.14]	.844
**Injury/disease characteristics**				
Mechanism	.09 [−.07–.25]	.255	.05 [−.10–.21]	.500
Nr Body regions affected	.11 [−.05–.26]	.176	.10 [−.06–25]	.220
Head (n,%)	.22 [.07–.37]	.006	.22 [.07–.37]	.006
Chest (n,%)	.01 [−.15–.16]	.950	−.06 [−.21–.10]	.495
Abdomen (n,%)	−.03 [−.19–.13]	.718	−.02 [−.18–.14]	.782
Limb (n,%)	−.03 [−.19–.13]	.688	.01 [−.15–.17]	.915
999-lactate sample (min)	.12 [−.04–.27]	.197	.07 [−.09–.23]	.422
**Indices of end-organ perfusion and oxygenation**				
Palpable radial pulse [y]	−.29 [−.42--.14]	<.001	−.17 [−.31--.01]	.038
SBP (mmHg)	−.31 [−.44--.16]	<.001	−.28 [−.42--.13]	.001
HR (bpm)	.21 [.06–.36]	.011	.19 [−.34–0.03]	.023
Shock index	.35 [.20–.48]	<.001	.34 [.19–.47]	<.001
First EtCO_2_ (kPa)	−.16 [−.31–.01]	.101	−.08 [−.24–.08]	.392
SpO_2_	−.24 [−.38--.08]	.005	−.19 [−.34--.03]	.024
**Treatments before HEMS**				
Circ and resp. support				
Adrenalin	.11 [−.04–.27]	.155	.09 [−.06–.25]	.254
IV fluids	.17 [.01–.31	.040	.16 [.01–.31]	.046
Pain relieving interventions				
IVP and/or morphine	−.24 [−.38--.09]	.003	−.19 [−.33--.03]	.021
IVP	−.18 [−.32--.02]	.027	−.17 [−.31--.01]	.039
Morphine	−.16 [−.31–.01]	.040	−.09 [−.24–.07]	.288
Reduction or splinting^a^	.10 [−.06–.25]	.227	.11 [−.04–.27]	.161
Haemostatic interventions				
Pelvic splint	.05 [−.10–.21]	.502	.05 [−.11–.20]	.576
Compression bandage or tourniquet	−.12 [−.27–.01	.126	−.07 [−.22–.09]	.384
TXA	.12 [−.04–.27]	.147	.09 [−.07–.24]	.278

*Footnote*: ^a^Manual reduction of fracture or dislocation and/or Kendrick and/or vacuum splint application. *SBP* Systolic blood pressure, *HR* Heart rate, *EtCO*_*2*_ End tidal CO_2_, *SpO*_*2*_ Percentage oxygen saturation, *IV* Intravenous, *IVP* Intravenous paracetamol, *TXA* Tranexamic acid

In multivariate analysis, SpO_2_, SI and IV analgetic administration prior to HEMS arrival remained independently associated with prehospital lactate, together explaining 17.7% of the variation in lactate levels (Table 3). Indices of end-organ perfusion and oxygenation (SpO2 and SI) were responsible for 15% of the variation in lactate levels. Sensitivity analysis revealed that within the subgroup of patients with a head injury (*n* = 76) SpO2 and SI predicted a similar percentage (17%) of the variation in lactate levels.

**Table 3:** Patient- and treatment characteristics of trauma patients associated with prehospital lactate levels (*n* = 156)

Variable	St ß	Adjusted R^2a^	*P*
First measured SpO2	−.271	.112	.001
Shock Index	.187	.039	.023
IVP or Morphine	−.184	.026	.024
Head Injury [y/n]	NA	NA	NA

*Footnote*. To avoid problems with co-linearity, Shock index was chosen over SBP, HR and palpable radial pulse in the regression model. ^a^denotes absolute increase in R^2^ when entered in the model. *NA* Did not enter final model, *SpO*_*2*_ Percentage oxygen saturation, *IVP* Intravenous paracetamol, *EtCO*_*2*_ End-tidal CO_2_, *TBI* Traumatic brain injury.

## Discussion

As expected, indices of end-organ perfusion- and oxygenation are associated with (elevated) lactate levels in prehospital trauma patients. However, these factors explained only 15% of the variation in prehospital lactate levels. Therefore, it is likely that other (independent) processes were responsible for lactate production- and clearance in these patients as well.

First, catecholamine release as a result of pain, stress or increased metabolic demand can result in lactate formation by activating intracellular cAMP, resulting in accelerated aerobic glycolysis [15, 16]. As trauma patients invariably have pain and/or stress, this is a likely contributor to pre-hospital lactate levels. We haven’t been able to quantify this directly in our study. However, it is well known that adequate analgesia blunts the physiological stress response and limits endogeneous catecholamine release, resulting in a decreased rate of glycolysis [17]. This is in agreement with the inverse relation between IV analgesic administration and lactate levels as found in this study.

Second, supportive treatments such as sodium chloride 0.9% or adrenaline administration, initiated before a blood sample for lactate measurement is drawn, may moderate HR, SBP, SI, and SpO_2_, and thereby influence tissue perfusion. The effect on lactate levels however, is more difficult to predict, as improved tissue perfusion may result in increased shuttling of lactate throughout the body [18]. This may have contributed to the (weak) positive association of fluid administration with elevated lactate levels as found in this study, although the effect of confounding by indication (sicker patients receiving more fluids) might have played a role as well.

Third, isolated injuries may result in elevated blood lactate levels, whereas their influence on indices of end organ perfusion- or oxygenation may be limited. Examples of these injuries are traumatic amputation [19] or isolated traumatic brain injury (TBI) [20]. Previous studies have shown that glia cells in the brain increase lactate production purposely in order to meet the increased metabolic demand of adjacent neurons during TBI [21]. This is in agreement with the association we found between head injured patients and (elevated) lactate levels in this study. As a significant interaction was also present between “head injury” and the administration of IV analgesics however, head injury did not contribute to the amount of explained variance in lactate levels in the multivariate model.

Furthermore, lactate levels measured at any point in time are not only the result of lactate production, but also of lactate clearance and utilization [15]. The liver takes up lactate from the blood, where it is reconverted to glucose in the Cori Cycle. Lactate can also be taken up by various tissues (brain, heart, muscle) and be directly utilized. Clearance is affected by various factors, including alcohol consumption (dose dependent decrease [22]) and (liver) tissue patency. Many of these are unknown to the clinicians caring for the patient in the prehospital situation.

In our study we observed that pre-hospital lactate levels not only represent end-organ perfusion, but also other processes, as the adrenergic response of the body to injuries and the adequacy of the initiated treatment(s). Elevated prehospital lactate levels should therefore not simply be considered as a marker of end organ hypoperfusion- or oxygenation. Although lactate levels were related to HR, SBP and SI in this study, correlation coefficients were low, especially compared to previously published in-hospital studies [23, 24]. The difference with in-hospital studies may be explained by the likelihood of the presence of a time lag between deterioration of vital signs and increased lactate formation and/or decreased lactate clearance. For in-hospital patients this argument may be less important, as sufficient time since the injury will have passed to ensure both lactate and vital signs are deranged.

Our study has some limitations, most of them inherent to the retrospective design. Firstly, the reported associations do not necessarily represent causality (as selection by indication might have been present for treatment factors as analgesics administration). This study should therefore mainly be regarded as hypothesis generating. Prospective studies are warranted to confirm our findings, especially regarding the effect of treatment factors on lactate, as there is an inherent risk of confounding by indication when considering the relation between lactate and treatment factors. Second, this study was done in a convenience sample of trauma patients in whom a lactate was measured, and cannot be extrapolated to the wider population of all prehospital trauma patients. Furthermore, although overall data completeness was good due to the use of our electronic patient record, there were missing data for some variables. Furthermore, as lactate samples were drawn before HEMS treatments (such as transfusion of blood products or RSI) were commenced, it is unclear how these advanced treatments would be related to lactate levels.

Finally, only single lactate measurements were available. Previous studies in other populations have shown that serial measurements improve not only prognostication, but might be helpful to guide treatment as well [25].

## Conclusion

Prehospital lactate levels are not solely associated with indices of end organ perfusion- and oxygenation. Injury type, treatments given on scene and many other (unmeasured) factors likely play an important role as well. This should be taken into account when lactate is used in clinical algorithms to guide prehospital triage or treatment.

## Supplementary information

**Additional file 1: Supplementary Table 1**. Standardised data collection proforma. **Supplementary Table 2**. Patients excluded**.**

## Data Availability

The datasets used and/or analysed during the current study are available from the corresponding author on reasonable request.

## Abbreviations

AAKSS: Air Ambulance Kent, surrey and Sussex
HEMS: Helicopter Emergency medical service
POCT: Point-of-care testing
SOP: Standard operating procedure

## References

[1] McGee S, Abernethy WB, Simel DL (1999). The rational clinical examination is this patient hypovolemic?. JAMA..

[2] Mullen M, Cerri G, Murray R, Talbot A, Sanseverino A, McCahill P (2014). Use of point-of-care lactate in the prehospital aeromedical environment. Prehosp Disaster Med.

[3] Brown JB, Lerner EB, Sperry JL, Billiar TR, Peitzman AB, Guyette FX (2016). Prehospital lactate improves accuracy of prehospital criteria for designating trauma activation level. J Trauma Acute Care Surg.

[4] Martín-Rodríguez Francisco, López-Izquierdo Raúl, Castro Villamor Miguel A., Mangas Iratxe Moro, del Brío Ibáñez Pablo, Delgado Benito Juan F., Martín Conty José L., Manzanares Jesús Álvarez, Mayo-Iscar Agustín, del Pozo Vegas Carlos (2019). Prognostic value of lactate in prehospital care as a predictor of early mortality. The American Journal of Emergency Medicine.

[5] Guyette FX, Meier EN, Newgard C, McKnight B, Daya M, Bulger EM (2015). A comparison of prehospital lactate and systolic blood pressure for predicting the need for resuscitative care in trauma transported by ground. J Trauma Acute Care Surg.

[6] Guyette F, Suffoletto B, Castillo J-L, Quintero J, Callaway C, Puyana J-C (2011). Prehospital serum lactate as a predictor of outcomes in trauma patients: a retrospective observational study. J Trauma Inj Infect Crit Care.

[7] Kushimoto S, Akaishi S, Sato T, Nomura R, Fujita M, Kudo D (2016). Lactate, a useful marker for disease mortality and severity but an unreliable marker of tissue hypoxia/hypoperfusion in critically ill patients. Acute Med Surg.

[8] Lewis CT, Naumann DN, Crombie N, Midwinter MJ (2016). Prehospital point-of-care lactate following trauma: a systematic review. J Trauma Acute Care Surg.

[9] Colon-Franco JM, Lo SF, Tarima SS, Gourlay D, Drendel AL, Brook LE (2017). Validation of a hand-held point of care device for lactate in adult and pediatric patients using traditional and locally-smoothed median and maximum absolute difference curves. Clin Chim Acta.

[10] Léguillier T, Jouffroy R, Boisson M, Boussaroque A, Chenevier-Gobeaux C, Chaabouni T (2018). Lactate POCT in mobile intensive care units for septic patients? A comparison of capillary blood method versus venous blood and plasma-based reference methods. Clin Biochem.

[11] Kruse O, Grunnet N, Barfod C (2011). Blood lactate as a predictor for in-hospital mortality in patients admitted acutely to hospital: a systematic review. Scand J Trauma Resusc Emerg Med.

[12] Shapiro NI, Howell MD, Talmor D, Nathanson LA, Lisbon A, Wolfe RE (2005). Serum lactate as a predictor of mortality in emergency department patients with infection. Ann Emerg Med.

[13] https://arc-w.nihr.ac.uk/Wordpress/wp-content/uploads/2017/06/Full-guidelines-for-Best-Practice-in-the-Ethics-and-Governance-of-Service-Evaluation-Final.pdf (Accessed 14 Jan 2020).

[14] von Elm E, Altman DG, Egger M, Pocock SJ, Gøtzsche PC, Vandenbroucke JP (2014). The strengthening the reporting of observational studies in epidemiology (STROBE) statement: guidelines for reporting observational studies. Int J Surg.

[15] Kraut JA, Madias NE (2014). Lactic acidosis. N Engl J Med.

[16] Levy B, Desebbe O, Montemont C, Gibot S (2008). Increased aerobic glycolysis through beta2 stimulation is a common mechanism involved in lactate formation during shock states. Shock.

[17] Gjedsted J, Buhl M, Nielsen S, Schmitz O, Vestergaard ET, Tønnesen E (2011). Effects of adrenalin on lactate, glucose, lipid and protein metabolism in the placebo controlled bilaterally perfused human leg. Acta Physiol (Oxf).

[18] Brooks GA (1986). The lactate shuttle during exercise and recovery. Med Sci Sports Exerc.

[19] Folkert IW, Sims CA, Pascual JL, Allen SR, Kim PK, Schwab CW (2015). Initial venous lactate levels in patients with isolated penetrating extremity trauma: a retrospective cohort study. Eur J Trauma Emerg Surg.

[20] Bouzat P, Oddo M (2014). Lactate and the injured brain: friend or foe?. Curr Opin Crit Care.

[21] Jha MK, Morrison BM (2018). Glia-neuron energy metabolism in health and diseases: new insights into the role of nervous system metabolic transporters. Exp Neurol.

[22] Dezman ZDW, Comer AC, Narayan M, Scalea TM, Hirshon JM, Smith GS (2016). Alcohol consumption decreases lactate clearance in acutely injured patients. Injury..

[23] Vandromme MJ, Griffin RL, Weinberg JA, Rue LW, Kerby JD (2010). Lactate is a better predictor than systolic blood pressure for determining blood requirement and mortality: could prehospital measures improve trauma triage?. J Am Coll Surg.

[24] Montoya KF, Charry JD, Calle-Toro JS, RamiroNúñez L, Poveda G (2015). Shock index as a mortality predictor in patients with acute polytrauma. J Acute Dis.

[25] Régnier MA, Raux M, Le Manach Y, Asencio Y, Gaillard J, Devilliers C (2012). Prognostic significance of blood lactate and lactate clearance in trauma patients. Anesthesiology..

