# Supplementary material for: Determinants of prehospital lactate in trauma patients: a retrospective cohort study
Source: BMC Emerg Med. 2020 Mar 11;20:18. doi: 10.1186/s12873-020-00314-1 (PMC7066760; doi:10.1186/s12873-020-00314-1)
Supplement: Supplementary file 1 — Additional file 1: Supplementary Table 1. Standardised data collection proforma. Supplementary Table 2. Patients excluded. [file 12873_2020_314_MOESM1_ESM.docx]

**Supplementary Digital content**

**Supplementary TABLE I: Standardised data collection proforma**

| **Mission timings** | 999 time |  |
| --- | --- | --- |
|  | Time lactate sample drawn |  |
| **Patient demographics** | Pt identification number |  |
|  | Age |  |
|  | Gender |  |
| **Patient injuries** | Mechanism of injury |  |
|  | Body regions involved |  |
| **Patient vital signs (on HEMS arrival)** | Heart rate |  |
|  | Systolic blood pressure |  |
|  | End-tidal CO_2_ |  |
|  | SpO_2_ |  |
| **Calculated variables** | Shock index |  |
| **Treatments initiated (pre-HEMS)** | Analgesia | IV paracetamol |
|  |  | Morphine |
|  |  | Fracture splintage |
|  | Haemostatic treatments | Fracture reduction |
|  |  | Pelvic binder |
|  |  | Tourniquet |
|  |  | Compression bandage |
|  |  | Tranexamic acid |
|  | Circulatory support | Intravenous fluids |
|  |  | Adrenaline |

**Supplementary TABLE II: Patients excluded.**

| **Reason for exclusion** | **n** |
| --- | --- |
| Hypoxia after dysrhythmia | 1 |
| Sepsis | 3 |
| Ruptured femoral aneurysm | 1 |
| Burns | 2 |
| Spontaneous subdural haematoma | 1 |
| Intoxication with alcohol or drugs | 3 |
| Hanging/ asphyxia | 1 |
| Seizure | 3 |
| Cardiac arrest | 2 |
| CVA | 1 |
